# A flexible-hydrogen interaction model for protein-ligand docking

**DOI:** 10.1186/1758-2946-4-S1-P14

**Published:** 2012-05-01

**Authors:** Angela M Henzler, Sascha Urbaczek, Benjamin Schulz, Matthias Rarey

**Affiliations:** 1 Center for Bioinformatics, University of Hamburg, Hamburg, 20146, Germany

## 

Although some docking methods accounting for protein flexibility exist, most large scale virtual screening approaches work with rigid protein models. A first step towards flexibility integration is the consideration of degrees of freedom resulting from hydrogens, especially, if involved in hydrogen bonding. To account for this type of flexibility, we present a flexible-hydrogen interaction model as part of a descriptor-based docking technique.

The model discretizes interaction spheres of rigid and flexible hydrogen-bond donors and acceptors as interaction spots. A spot has an associated interaction direction which indicates hydrogen or lone pair orientation, and thus, the potential location of a hydrogen-bond counterpart. This new flexible-hydrogen interaction model is combined with a novel approach to describe hydrophobic contacts. Both are introduced in our descriptor-based docking approach named TrixX [1]. TrixX handles ligand flexibility by applying a conformer ensemble approach [2]. The latter allows for the use of efficient indexing techniques upon virtual screening. The discretized, flexible-hydrogen model proposes potential hydrogen and lone pair positions. However, these proposals may still slightly differ from their actual location which can be only determined in presence of pose and active site, i. e., after the docking stage. In order to grant a thorough assessment of hydrogen bonds, thereby, the predicted poses are forwarded to an efficient post-optimization of the hydrogen-bond network. It optimally aligns hydrogens, identifies favorable tautomeric and protonation states, and evaluates the predicted pose [3].

Redocking of the Astex Diverse Set [4] shows that the described docking method produces results in good agreement with co-crystalized ligand structures. Several case studies using different levels of discretization and post-optimization, illustrate the influence of our presented procedures in ligand placement and scoring. The studies highlight the impact of flexible hydrogens and lone pairs during docking and confirm the introduction of our flexible hydrogen model.
